# Search for topological defect dark matter with a global network of optical magnetometers

**DOI:** 10.1038/s41567-021-01393-y

**Published:** 2021-12-07

**Authors:** Samer Afach, Ben C. Buchler, Dmitry Budker, Conner Dailey, Andrei Derevianko, Vincent Dumont, Nataniel L. Figueroa, Ilja Gerhardt, Zoran D. Grujić, Hong Guo, Chuanpeng Hao, Paul S. Hamilton, Morgan Hedges, Derek F. Jackson Kimball, Dongok Kim, Sami Khamis, Thomas Kornack, Victor Lebedev, Zheng-Tian Lu, Hector Masia-Roig, Madeline Monroy, Mikhail Padniuk, Christopher A. Palm, Sun Yool Park, Karun V. Paul, Alexander Penaflor, Xiang Peng, Maxim Pospelov, Rayshaun Preston, Szymon Pustelny, Theo Scholtes, Perrin C. Segura, Yannis K. Semertzidis, Dong Sheng, Yun Chang Shin, Joseph A. Smiga, Jason E. Stalnaker, Ibrahim Sulai, Dhruv Tandon, Tao Wang, Antoine Weis, Arne Wickenbrock, Tatum Wilson, Teng Wu, David Wurm, Wei Xiao, Yucheng Yang, Dongrui Yu, Jianwei Zhang

**Affiliations:** 1 Helmholtz-Institut Mainz, GSI Helmholtzzentrum für Schwerionenforschung, Darmstadt, Germany; 2 Johannes Gutenberg-Universität Mainz, Mainz, Germany; 3 Centre for Quantum Computation and Communication Technology, Research School of Physics, The Australian National University, Acton, ACT Australia; 4 Department of Physics, University of California at Berkeley, Berkeley, CA USA; 5 Department of Physics, University of Nevada, Reno, NV USA; 6 Computational Research Division, Lawrence Berkeley National Laboratory, Berkeley, CA USA; 7 Institute for Quantum Science and Technology (IQST), 3rd Institute of Physics, and Max Planck Institute for Solid State Research, Stuttgart, Germany; 8 Institute of Physics Belgrade, University of Belgrade, Belgrade, Serbia; 9 Physics Department, University of Fribourg, Fribourg, Switzerland; 10 State Key Laboratory of Advanced Optical Communication Systems and Networks, Department of Electronics, and Center for Quantum Information Technology, Peking University, Beijing, China; 11 Department of Precision Machinery and Precision Instrumentation, University of Science and Technology of China, Hefei, People’s Republic of China; 12 Department of Physics and Astronomy, University of California, Los Angeles, CA USA; 13 Department of Physics, California State University–East Bay, Hayward, CA USA; 14 Center for Axion and Precision Physics Research, IBS, Daejeon, Republic of Korea; 15 Department of Physics, KAIST, Daejeon, Republic of Korea; 16 Twinleaf LLC, Plainsboro, NJ USA; 17 Hefei National Laboratory for Physical Sciences at the Microscale, University of Science and Technology of China, Hefei, People’s Republic of China; 18 Institute of Physics, Jagiellonian University, Kraków, Poland; 19 Department of Physics and Astronomy, Oberlin College, Oberlin, OH USA; 20 School of Physics and Astronomy, University of Minnesota, Minneapolis, MN USA; 21 William I. Fine Theoretical Physics Institute, School of Physics and Astronomy, University of Minnesota, Minneapolis, MN USA; 22 Leibniz Institute of Photonic Technology, Jena, Germany; 23 Department of Physics and Astronomy, Bucknell University, Lewisburg, PA USA; 24 Department of Physics, Princeton University, Princeton, NJ USA; 25 Technische Universität München, Garching, Germany; 26 Present Address: Department of Physics and Astronomy, University of Waterloo, Waterloo, Canada; 27 Present Address: JILA, NIST and Department of Physics, University of Colorado, Boulder, CO USA; 28 Present Address: Department of Physics, Harvard University, Cambridge, MA USA

**Keywords:** Atomic and molecular physics, Dark energy and dark matter, Particle physics

## Abstract

Ultralight bosons such as axion-like particles are viable candidates for dark matter. They can form stable, macroscopic field configurations in the form of topological defects that could concentrate the dark matter density into many distinct, compact spatial regions that are small compared with the Galaxy but much larger than the Earth. Here we report the results of the search for transient signals from the domain walls of axion-like particles by using the global network of optical magnetometers for exotic (GNOME) physics searches. We search the data, consisting of correlated measurements from optical atomic magnetometers located in laboratories all over the world, for patterns of signals propagating through the network consistent with domain walls. The analysis of these data from a continuous month-long operation of GNOME finds no statistically significant signals, thus placing experimental constraints on such dark matter scenarios.

## Abstract (web summary)

A search for transient dark matter in the form of domain walls of axion-like particles finds no statistically significant signal. This places constraints on our theoretical understanding of such scenarios.

## Main

The nature of dark matter—an invisible substance comprising over 80% of the mass of the Universe^1,2^—is one of the most profound mysteries of modern physics. Although evidence for the existence of dark matter comes from its gravitational interactions, unravelling its nature likely requires observing non-gravitational interactions between dark matter and ordinary matter^3^. One of the leading hypotheses is that dark matter consists of ultralight bosons such as axions^4^ or axion-like particles (ALPs)^5–7^. Axions and ALPs arise from spontaneous symmetry breaking at an unknown energy scale *f*_SB_, which—along with their mass *m*_a_—determines many of their physical properties.

ALPs can manifest as stable, macroscopic field configurations in the form of topological defects^8–10^ or composite objects bound together by self-interactions such as boson stars^11,12^. Such ALP field configurations could concentrate the dark matter density into many distinct, compact spatial regions that are small compared with the Galaxy but much larger than the Earth. In such scenarios, Earth-bound detectors would only be able to measure signals associated with dark matter interactions on occasions when the Earth passes through such a dark matter object. It turns out that there is a wide range of parameter space—consistent with observations—for which such dark matter objects can have the required size and abundance such that the characteristic time between encounters could be of the order of one year or less^9,10,12^. This opens up the possibility of searches with terrestrial detectors. Here we present the results of such a search for ALP domain walls, a class of topological defects that can form between regions of space with different vacua of an ALP field^8,9^. We note that although some models suggest that axion domain walls cannot survive to the present epoch^13–15^, there do exist a number of ALP models demonstrating the theoretical possibility that ALP domain walls or composite dark matter objects with similar characteristics^12,16,17^ can survive to modern times^18–20^ and have the characteristics of cold dark matter^9,10,21^.

Since ALPs can interact with atomic spins^3^, the passage of Earth through an ALP domain wall affects atomic spins similar to a transient magnetic-field pulse^9,12^. Considering a linear coupling between the ALP field gradient ${{{\bf{\nabla }}}} a ({{{\bf{r}}}},t)$ and atomic spin **S**, the interaction Hamiltonian can be written as

1 $${H}_{{{{\rm{lin}}}}}=-{(\hslash c)}^{3/2}\frac{\xi }{{f}_{{{\rm{SB}}}}}\frac{{{{\bf{S}}}}}{\parallel S\parallel }\cdot {{{\bf{\nabla }}}} a ({{{\bf{r}}}},t)\,,$$

where *ℏ* is the reduced Planck’s constant, *c* is the speed of light, **r** is the position of spin, *t* is time, and *f*_SB_/*ξ* ≡ *f*_int_ is the coupling constant in units of energy described with respect to the symmetry-breaking scale *f*_SB_ (ref. ^22^); here *ξ* is unitless. In most theories, the coupling constants *f*_int_ describing the interaction between standard model fermions and the ALP field are proportional to *f*_SB_; however, *f*_int_ can differ between electrons, neutrons and protons by model-dependent factors that can be substantial^3,5^.

Analogous to equation (1), the Zeeman Hamiltonian describing the interaction of magnetic field **B** with atomic spin **S** can be written as

2 $${H}_{{{{\rm{Z}}}}}=-\gamma {{{\bf{S}}}}\cdot {{{\bf{B}}}}\,,$$

where *γ* is the gyromagnetic ratio. Since equations (1) and (2) have the same structure, the gradient of the ALP field—even though it couples to the particle spin rather than the magnetic moment—can be treated as a ‘pseudo-magnetic field’ as it causes energy shifts of Zeeman sublevels. An important distinction between the ALP-spin interaction (equation (1)) and the Zeeman interaction (equation (2)) is that although *γ* tends to scale inversely with the fermion mass, no such scaling of the ALP-spin interaction is expected^3^.

The amplitude, direction and duration of the pseudo-magnetic-field pulse associated with the transit of the Earth through an ALP domain wall depends on many unknown parameters such as the energy density stored in the ALP field, coupling constant *f*_int_, thickness of the domain wall, and relative velocity **v** between Earth and the domain wall. The dynamical parameters, such as the velocities of dark matter objects, are expected to randomly vary from encounter to encounter. We assume that they are described by the standard halo model for virialized dark matter^23^. Furthermore, the abundance of domain walls in the Galaxy is limited by physical constants, namely, *m*_a_ and *f*_SB_, as these determine the energy contained in the wall, and the total energy of all the domain walls is constrained by estimates of the local dark matter density^24^. The expected temporal form of the pseudo-magnetic-field pulse can depend on the theoretical model describing the ALP domain wall as well as particular details of the terrestrial encounter (such as the orientation of Earth). The relationships between these parameters and characteristics of the pseudo-magnetic-field pulses searched for in our analysis are discussed in Supplementary Section II and other studies^9,12,22^.

**Fig. 1:**
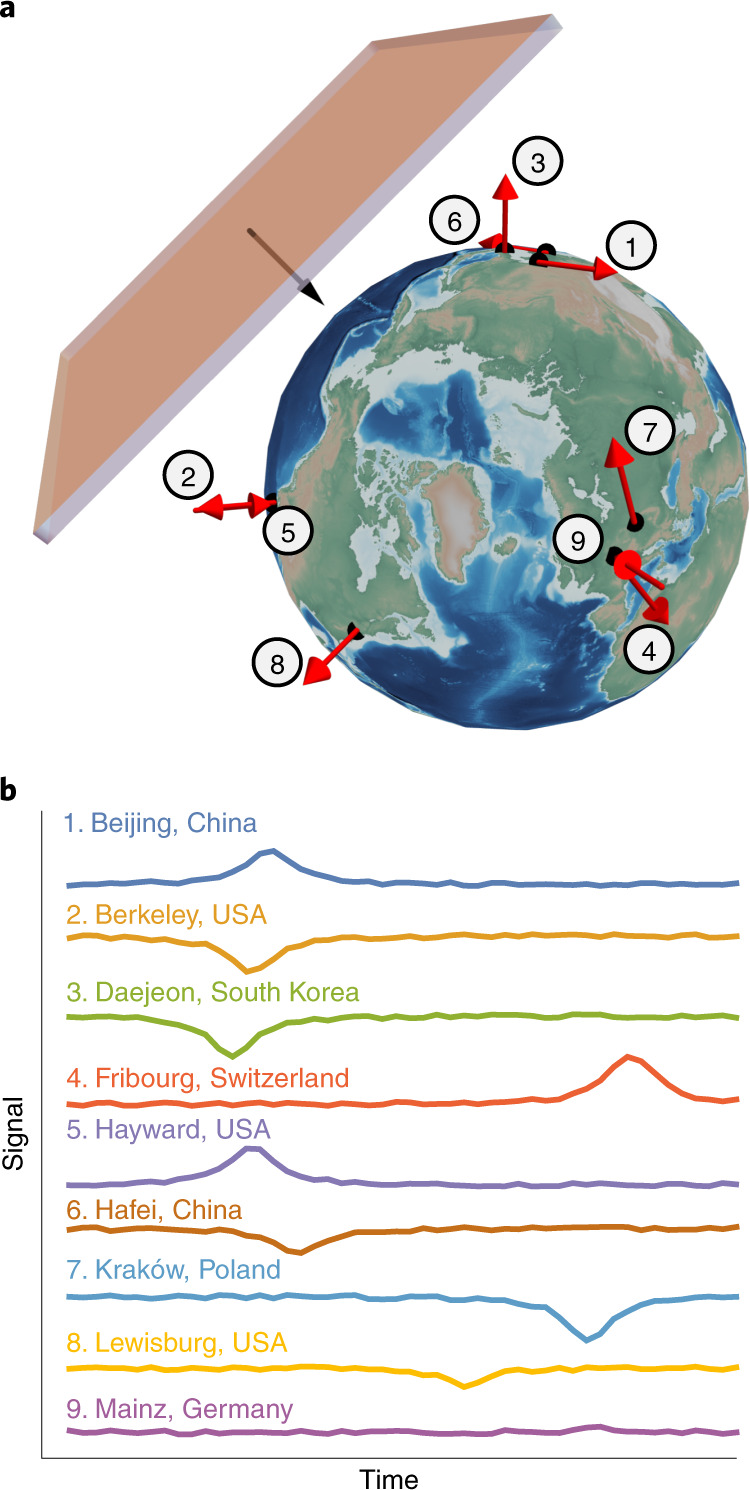
Visualization of an ALP domain-wall crossing. **a**, Image showing the Earth together with the position and sensitive axes of the GNOME magnetometers during Science Run 2. Position and sensitive axes are show as red arrows. The crossing direction of the domain wall is represented as a black arrow (Extended Data Table 1). **b**, Simulation of the signals expected to be observed from a domain-wall crossing at the different magnetometers comprising the network.

The global network of optical magnetometers for exotic (GNOME) physics searches is a worldwide network searching for correlated signals heralding beyond-the-standard-model physics that currently comprises more than a dozen optical atomic magnetometers, with stations (each with a magnetometer and supporting devices) in Europe, North America, Asia, the Middle East and Australia. A schematic of a domain-wall encounter with GNOME is shown in Fig. 1. The measurements from the magnetometers are recorded with custom data-acquisition systems^25^; synchronized to the global positioning system (GPS) time; and uploaded to servers located in Mainz, Germany, and Daejeon, South Korea. Descriptions of the operational principles and characteristics of GNOME magnetometers are presented in Methods, Extended Data Table 1, and ref. ^26^.

The active field sensor at the heart of every GNOME magnetometer is an optically pumped and probed gas of alkali atoms. Magnetic fields are measured by variations in the Larmor spin precession of the optically polarized atoms. The vapour cells containing the alkali atoms are placed inside multilayer magnetic-shielding systems that reduce background magnetic noise by orders of magnitude^27^ despite retaining sensitivity to exotic spin couplings between ALP dark matter and atomic nuclei.

If the ALP field only couples to electron spins, interactions between the ALP field and magnetic shield will reduce the ALP-induced signal amplitudes in each magnetometer by roughly the magnetic shielding factors of 10^6^–10^7^, as discussed in ref. ^28^. Therefore, in the present work, we only consider interactions between ALP fields and atomic nuclei. Since all the GNOME magnetometers presently use atoms whose nuclei have a valence proton, the signal amplitudes measured by GNOME due to an ALP-spin interaction are proportional to the relative contribution of proton spin to nuclear spin (as discussed in Supplementary Section II and ref. ^29^). This pattern of signal amplitudes (equation (1)) can be characterized by a pseudo-magnetic field *B*_*j*_ measured with sensor *j*:

3 $${B}_{j}=\frac{{\sigma }_{j}{\eta }_{j}}{{g}_{\mathrm{F},j}}{{{{\mathcal{B}}}}}_{{{\rm{p}}}}\,,$$

where

4 $${{{{\mathcal{B}}}}}_{{{\rm{p}}}}({{{\bf{r}}}},t)={\left(\hslash c\right)}^{3/2}\frac{2\xi }{{\mu }_{\mathrm{B}}{f}_{{{\rm{SB}}}}}{{{\bf{\nabla }}}} a({{{\bf{r}}}},t)$$

is the normalized pseudo-magnetic field describing the effect of the ALP domain wall on proton spins and *μ*_B_ is the Bohr magneton. The ratio between the Landé *g*-factor and the effective proton spin (*g*_F,*j*_/*σ*_*j*_) accounts for the specific proton-spin coupling in the respective sensor. This ratio depends on the atomic and nuclear structure in addition to details of the magnetometry scheme (Supplementary Section II). Since each GNOME magnetometer measures the projection of the field along a particular sensitive axis, the factor *η*_*j*_ is introduced to account for directional sensitivity. This factor, given by the cosine of the angle between ${{{{\mathcal{B}}}}}_{{{\rm{p}}}}$ and the sensitive axes, takes on values between +1 and –1.

In spite of the unknown properties of a particular terrestrial encounter with an ALP domain wall, GNOME measures a recognizable global pattern of the associated amplitudes of the pseudo-magnetic-field pulse described by equation (3), as illustrated in Fig. 1b. The associated pseudo-magnetic-field pulses would point along a common axis, have the same duration and exhibit a characteristic timing pattern. The data-analysis algorithm used in the present work to search for ALP domain walls is described in Methods and ref. ^30^. The algorithm searches for a characteristic signal pattern across GNOME, having properties consistent with the passage of Earth through an ALP domain wall. Separate analyses to search for transient oscillatory signals associated with boson stars^12^ and bursts of exotic low-mass fields from cataclysmic astrophysical events^31^ are presently underway.

Here we report the results of a dark matter search with GNOME: a search for transient couplings of atomic spins to macroscopic dark matter objects, thereby demonstrating the ability of GNOME to explore the parameter space previously unconstrained by direct laboratory experiments. Searches for macroscopic dark matter objects based on similar ideas were carried out using atomic clock networks^10,23,32,33^, and there are a number of experimental proposals utilizing other sensor networks^34–37^. All these networks are sensitive to bosonic dark matter with a scalar coupling to standard model particles^3^. GNOME is sensitive to a different class of dark matter: bosons with pseudo-scalar couplings to standard model particles. Pseudo-scalar bosonic dark matter generally produces no observable effects in clock networks^3^, but it does couple to atomic spins via the interaction described by equation (1). Thus, GNOME is sensitive to a distinct—so far, mostly unconstrained—class of interactions compared with other sensor networks.

## Search for ALP domain-wall signatures

**Extended Data Fig. 1:**
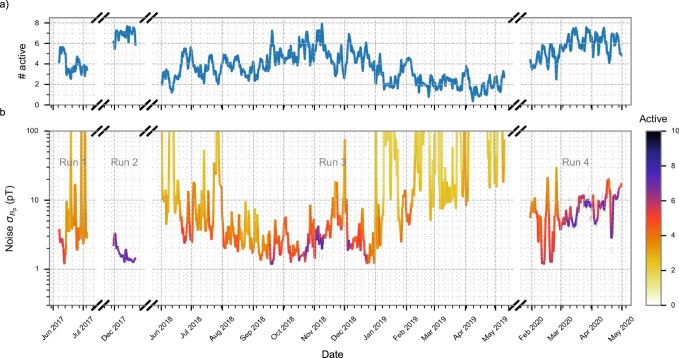
Summary of the GNOME performance during the four Science Runs from 2017 to 2020. The raw magnetometer data are averaged for 20 s and their standard deviation is calculated over a minimum of one and a maximum of two hours segments depending on the availability of continuous data segments. For each binned point, the combined network noise considering the worst case domain-wall crossing direction is evaluated as defined in Ref. ^30^. (a) One-day rolling average of the number of active sensors. (b) Multi-colored solid line represents the one-day rolling average of the combined network noise and the multi-colored dashes show the noise of the individual sampled segments. The data are preprocessed with the same filters used for the analysis. The number of magnetometers active is indicated by the color of the line and dashes. Source data

There have been four GNOME science runs between 2017 and 2020, as discussed in Methods. Here we analyse the data from Science Run 2, which had comparatively good overall noise characteristics and consistent network operation (as shown in Extended Data Fig. 1). Nine magnetometers took part in Science Run 2 that spanned from 29 November 2017 to 22 December 2017. The characteristics of the magnetometers are summarized in Extended Data Table 1.

Before the data are searched for evidence of domain-wall signatures, they are preprocessed by applying a rolling average, high-pass filters, and notch filters to the raw data. The averaging process enhances the signal-to-noise ratio for certain pulse durations, avoids complications arising from different magnetometers having different bandwidths, and reduces the amount of data to be analysed. The high-pass and notch filters reduce the effects of long-term drifts and noisy frequency bands. We refer to the filtered and rolling-averaged dataset as the ‘search data.’

The search data are examined for the evidence of collective signal patterns corresponding to planes with uniform, non-zero thickness, crossing Earth at constant velocities. The imprinted pattern of amplitudes depends on the domain-wall-crossing velocity^30^. We assume that the domain-wall-velocity probability density function follows the standard halo model for virialized dark matter. The signature of a domain wall crossing the magnetometer network depends on the component of the relative velocity between the domain wall and the Earth that is perpendicular to the domain-wall plane, **v**_⊥_. A lattice of points in the velocity space is constructed such that the search algorithm covers 97.5% of the velocity probability density function. The algorithm scans over the velocity lattice and, for every velocity, the data from each magnetometer are appropriately time-shifted so that the signals in different magnetometers from a hypothetical domain-wall crossing with the given velocity occur at the same time. For each velocity and at each measurement time, the amplitudes measured by each magnetometer are fit to the ALP domain-wall-crossing model described in ref. ^30^. As a result, estimations for signal magnitude and domain-wall direction, along with associated uncertainties, are obtained for each measurement time and all the lattice velocities. The magnitude-to-uncertainty ratio of an event is given by the ratio between the signal magnitude and its associated uncertainty.

**Extended Data Fig. 2:**
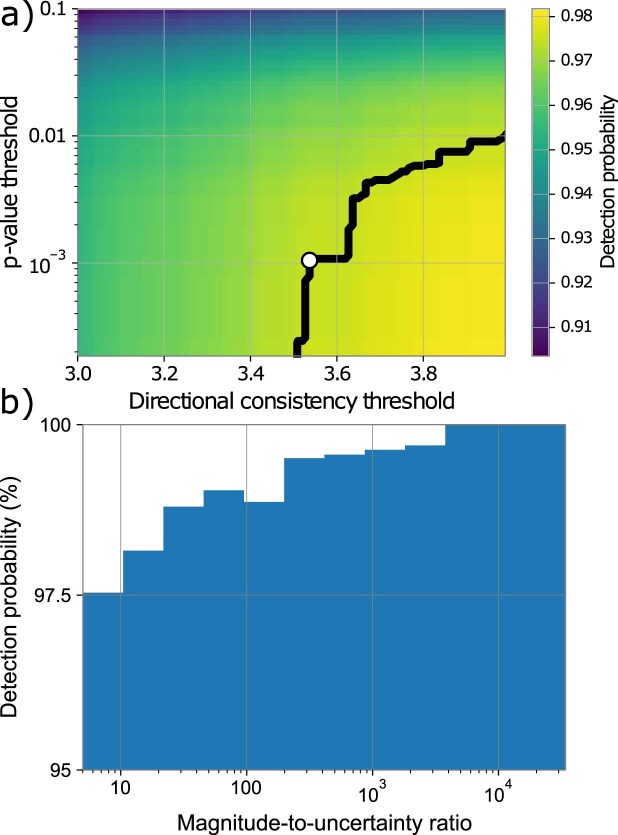
Summary of the true-positive analysis results. (a) shows the probability of detecting a domain-wall-crossing event with randomized parameters (as discussed in the text) as a function of p-value and directional-consistency thresholds. The inserted events have a magnitude-to-uncertainty ratio between 5 and 10. The black line indicates the combination of parameters corresponding to a 97.5% detection probability. The white dot indicates the particular thresholds chosen for the analysis. (b) Shows the mean detection probability reached for different magnitude-to-uncertainty ratios for the chosen thresholds.

The search algorithm uses two different tests to evaluate if a given event is likely to have been produced by an ALP domain-wall crossing: a domain-wall model test and a directional-consistency test^30^. The domain-wall model test evaluates whether the event amplitudes measured by the GNOME magnetometers match the signal amplitudes predicted by the ALP domain-wall-crossing model, and is quantified by the *P*-value, as discussed in Methods and ref. ^30^. The directional-consistency test checks the agreement between the direction of the scanned velocity and the estimated domain-wall direction, and is quantified by the angle between the two directions normalized by the angle between the adjacent lattice velocities. The thresholds on these tests are chosen to guarantee an overall detection efficiency *ϵ* ≥ 95% for the search algorithm, considering both incomplete velocity lattice coverage and detection probability (Extended Data Fig. 2).

**Fig. 2:**
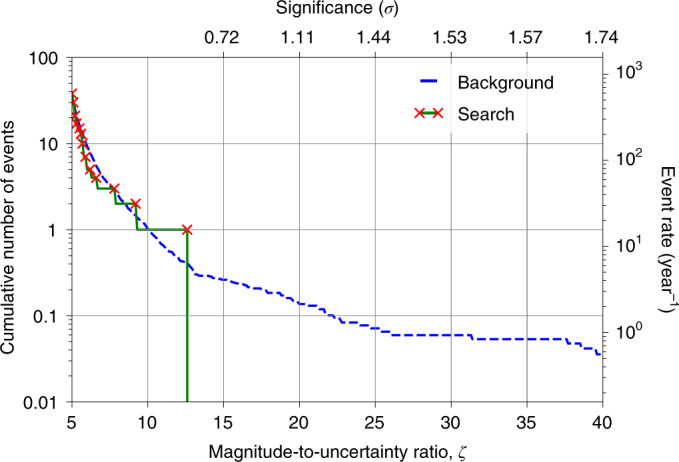
Significance of the search events. The blue dashed line represents the cumulative number of events expected from the background in the 23 days of data from Science Run 2. Here 10.7 years of time-shuffled data are used to evaluate the background. Such a duration is an arbitrary choice, but it is sufficiently long to characterize the background. The number of candidate events measured in the background data is re-scaled to the duration of Science Run 2. The solid green line represents the cumulative number of events measured in Science Run 2. The red crosses indicate the magnitude-to-uncertainty ratio at which new events are found in the search data. The upper axis indicates the statistical significance in units of Gaussian standard deviations of finding one event in the search data. The significance is given by the probability of detecting one or more background events at a magnitude-to-uncertainty ratio above that of the candidate event (equation (5)). The right axis shows the normalized number of events over a period of a year. The event with the greatest magnitude-to-uncertainty ratio is found at 12.6. Source data

The search data are analysed for domain-wall encounters using the algorithm presented in ref. ^30^. The cumulative distribution of candidate events as a function of their magnitude-to-uncertainty ratio is shown as the solid green line in Fig. 2. The candidate event in the search data with the largest magnitude-to-uncertainty ratio (namely, 12.6) had a significance of less than one sigma. Therefore, we find no evidence of an ALP domain-wall crossing during Science Run 2. Rare domain-wall-crossing events that produce signals below a magnitude-to-uncertainty ratio of 12.6 are indistinguishable from the background. Therefore, we base constraints on the ALP parameters on the absence of any detection above the ‘loudest event’ in a manner similar to that described, for example, in ref. ^38^.

**Fig. 3:**
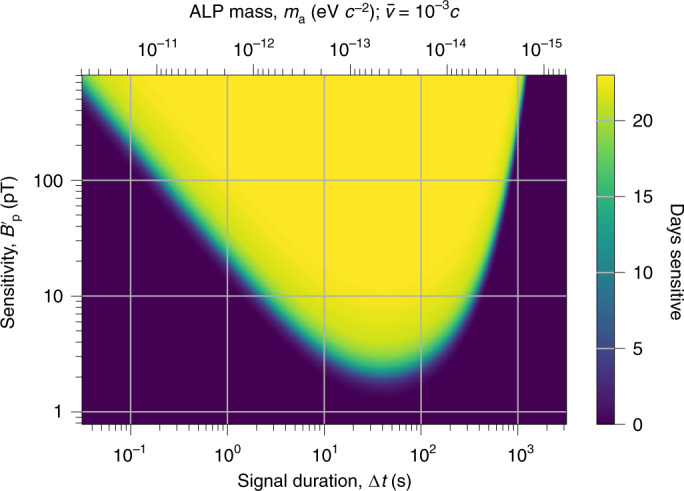
Sensitivity of the GNOME network to domain walls. Amount of time *T*, indicated in colour, for which GNOME had a normalized pseudo-magnetic-field-magnitude sensitivity above ${{{{\mathcal{B}}}}}_{{{{\rm{p}}}}}^{\prime}$ (that is, the domain wall would induce a magnitude-to-uncertainty ratio of at least one) for domain walls with a given duration Δ*t* (defined as the FWHM of a Lorentzian signal) throughout Science Run 2. The upper axis shows the range of ALP masses to which GNOME is sensitive (equation (9)). The characteristic shape of the sensitive region is a result of the filtering and averaging of the raw data, as described in Methods. Averaging reduces the sensitivity of the search data to short pulse durations and high-pass filtering suppresses sensitivity to long Δ*t*. The sensitivity of GNOME varies in time with changes in the number of active GNOME magnetometers recording data and their background noise. Only the worst-case direction is considered. The plot assumes the parameters of the analysis: 20 s averaging time, 1.67 mHz first-order zero-phase Butterworth filter, and 50 and 60 Hz zero-phase notch filters with a quality factor of 60. Source data

To evaluate the domain-wall characteristics excluded by this result, the observable domain-wall-crossing parameters above a magnitude-to-uncertainty ratio of 12.6 during Science Run 2 are determined. GNOME has non-uniform directional sensitivity^30^; we conservatively estimate the network sensitivity assuming the domain wall comes from the least-sensitive direction. Figure 3 shows the active time *T*(${{\Delta }}t,{{{{\mathcal{B}}}}}_{{{{\rm{p}}}}}^{\prime}$), that is, how long the network was sensitive to domain walls as a function of sensitivity of the pseudo-magnetic-field magnitude, ${{{{\mathcal{B}}}}}_{{{{\rm{p}}}}}^{\prime}$, and pulse duration, Δ*t*. A signal with pseudo-magnetic-field magnitude ${{{{\mathcal{B}}}}}_{{{\rm{p}}}}$ produces a magnitude-to-uncertainty ratio of $\zeta ={{{{\mathcal{B}}}}}_{{{\rm{p}}}}/{{{{\mathcal{B}}}}}_{{{{\rm{p}}}}}^{\prime}$. The active time, *T*(${{\Delta }}t,{{{{\mathcal{B}}}}}_{{{{\rm{p}}}}}^{\prime}$), can be used to constrain the ALP domain-wall parameter space, as discussed in Supplementary Section II.

If one assumes a probability distribution for the number of domain-wall encounters, an upper bound on the rate *R*_*C*_ of such encounters can be calculated with confidence level *C*. We assume a Poisson probability distribution for the domain-wall crossings. Since the excess number of events in the search data compared with the background data was not statistically significant, the upper bound on the observable rate is given by the probability of measuring no events during the effective time^38^. Note that since *T* depends on the parameters of the domain-wall crossing, our constraint on the observed rate depends on the ALP properties. We choose the confidence level to be *C* = 90%.

## Constraints on ALP domain walls

Analysis of the GNOME data did not find any statistically significant excess of events above the background during Science Run 2 that could point to the existence of ALP domain walls, as shown in Fig. 2. The expected rate of domain-wall encounters (*r*) depends on the ALP mass (*m*_a_), domain-wall energy density in the Milky Way (*ρ*_DW_), typical relative domain-wall speed $(\bar{v})$ and symmetry-breaking scale (*f*_SB_). The region of parameter space to which GNOME is sensitive is defined by the ALP parameters expected to produce signals above the magnitude-to-uncertainty ratio of 12.6 with rates *r* ≥ *R*_90%_ during Science Run 2 (Fig. 3). Based on the null result of our search, the sensitive region is interpreted as the excluded ALP parameter space.

The ALP parameters and the phenomenological parameters describing the ALP domain walls in our Galaxy, namely, thickness Δ*x*, surface tension or energy per unit area *σ*_DW_, and the average separation *L*, can be related through the ALP domain-wall model described elsewhere^9,22^. A full derivation of how the observable parameters are related to the ALP parameters is given in Supplementary Section II.

**Fig. 4:**
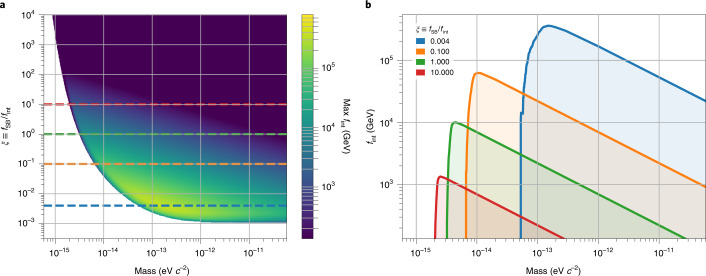
Bounds on the ALP parameter space. The bounds are drawn from the presented analysis of Science Run 2 with 90% confidence level. Relationship between the parameters from ALP theory and measured quantities is discussed in Supplementary Section II. **a**, In colour, upper bound on the interaction scale for axion–nucleon coupling, *f*_int_, to which GNOME was sensitive as a function of *m*_a_ and the ratio between symmetry-breaking and interaction scales (*ξ* ≡ *f*_SB_/*f*_int_). The dashed horizontal lines highlight the cross-section used in **b** with the respective colour. **b**, Cross-sections of the excluded parameter volume in **a** for different *ξ* ratios. We note that the domain walls may not be the only form of dark matter; therefore, *ρ*_DW_ < 0.4 GeV cm^–3^. If the domain-wall energy density is substantially smaller, this would affect the bounds shown here. Source data

The coloured region in Fig. 4a describes the symmetry-breaking scales up to which GNOME was sensitive with 90% confidence. The parameter space is spanned by ALP mass, maximum symmetry-breaking scale, and ratio between the symmetry-breaking scale and coupling constant. The shape of the sensitive area shown in Fig. 4a is determined by the event with the largest magnitude-to-uncertainty ratio and the characteristics of preprocessing applied to the raw data.

Figure 4b shows the various cross sections for different ratios between the symmetry-breaking scale and the coupling constant, as indicated by the dashed lines in Fig. 4a. The upper bound of *f*_SB_ that can be observed by the network is shown in Fig. 4b for different values of *ξ* ≡ *f*_SB_/*f*_int_. Because ${{{{\mathcal{B}}}}}_{{{\rm{p}}}}\propto {m}_{\mathrm{a}}$ (Supplementary equation (10) in Supplementary Section II), there is a sharp cutoff for low ALP mass where the corresponding field magnitude falls below the network sensitivity. Even though ${{{{\mathcal{B}}}}}_{{{\rm{p}}}}$ increases for large *m*_a_, the mean rate of domain-wall encounters decreases with increasing mass (equations (11) and (12)). Correspondingly, the upper limit for the symmetry-breaking scale *f*_SB_ is $\propto 1/\sqrt{{m}_{\mathrm{a}}}$. Given that no events were found, the sensitive region of the ALP domain-wall parameter space during Science Run 2 can be excluded.

Our experiment explores the ALP parameter space up to *f*_int_ ≈ 4 × 10^5^ GeV (Fig. 4). This goes beyond that excluded by previous direct laboratory experiments searching for ALP-mediated exotic pseudo-scalar interactions between protons that have shown that *f*_int_ ≳ 300 GeV over the ALP mass range probed by GNOME^39^. Although astrophysical observations suggest that *f*_int_ ≳ 2 × 10^8^ GeV, there are a variety of scenarios in which such astrophysical constraints can be evaded^40,41^. The parameter space for *f*_int_ and *m*_a_ explored in this search is well outside the typical predictions for axions in quantum chromodynamics^42,43^. However, for ALPs, a vast array of possibilities for the generation of ALP masses and couplings are opened by a variety of beyond-the-standard-model theories, meaning that the values of *f*_int_ and *m*_a_ explored in our search are theoretically possible^44,45^.

Future work of the GNOME collaboration will focus on both upgrades to our experimental apparatus and new data-analysis strategies. One of our key goals is to improve the overall reliability and duration of continuous operation of GNOME magnetometers. The intermittent operation of some magnetometers due to technical difficulties during Science Runs 1–3 made it difficult to search for signals persisting for ≳1 h. Additionally, magnetometers varied in their bandwidths and reliability, as well as stability of their calibration. These challenges were addressed in Science Run 4 through a variety of magnetometer upgrades and instituting daily worldwide test and calibration pulse sequences. However, GNOME suffered disruptions due to the COVID-19 pandemic. We plan to carry out Science Run 5 in 2021 to take full advantage of the improvements. Furthermore, by upgrading to noble-gas-based comagnetometers^46,47^ for future science runs (advanced GNOME), we expect to considerably improve the sensitivity to ALP domain walls. Additionally, GNOME data can be searched for other signatures of physics beyond the standard model, such as boson stars^12^, relaxion halos^48^ and bursts of exotic low-mass fields from black-hole mergers^31^.

In terms of the data-analysis algorithm used to search for ALP domain walls, recent studies^49^ have considered a possible back-action that the Earth may have on a domain wall when certain interactions are important, namely, up-to-quadratic coupling terms between a scalar field and fermions. In contrast to another study^49^, the present work analyses a completely different interaction, namely, a linear coupling between a pseudo-scalar field and fermion spins, which produces no major back-action effect. Regardless, it would be worthwhile to consider interactions generating similar back-action effects of the Earth on domain walls and the ALP field in later analysis. Further, in future work, we aim to improve the efficiency of the scan over the velocity lattice. The number of points in the velocity lattice to reliably cover a fixed fraction (for example, 97.5%) of the ALP-velocity probability distribution grows as (Δ*t*)^–3^ (where Δ*t* is given by equation (9)). This makes the algorithm computationally intensive. We are investigating a variety of analysis approaches, such as machine-learning-based algorithms, to address these issues.

## Methods

GNOME consists of over a dozen optical atomic magnetometers, each enclosed within a multilayer magnetic shield, distributed around the world^27^. GNOME magnetometers are based on a variety of different atomic species, optical transitions and measurement techniques: some are frequency- or amplitude-modulated nonlinear magneto-optical rotation magnetometers^50,51^, some are radio-frequency-driven optical magnetometers^26^, whereas others are spin-exchange-relaxation-free magnetometers^52^. A detailed description and characterization of six GNOME magnetometers are given in ref. ^26^. A summary of the properties of the GNOME magnetometers active during Science Run 2 is presented in Extended Data Table 1.

Each GNOME station is equipped with auxiliary sensors, including accelerometers, gyroscopes and unshielded magnetometers, to measure local perturbations that could mimic a dark matter signal. Suspicious data are flagged^26^ and discarded during the analysis.

The number of active GNOME magnetometers during the four science runs and the combined network noise, as defined in ref. ^30^, are shown as a function of time in Extended Data Fig. 1. Although Science Run 4 was carried out over a longer period of time than Science Run 2, it featured poorer noise characteristics and consistency of operation compared with Science Run 2. Since many GNOME stations underwent upgrades in 2018 and 2019, further characterization of the data from Science Run 4 is needed, and the results will be presented in future work. The number of active magnetometers during Science Runs 1 and 3 was often less than four, which in insufficient to characterize a domain-wall crossing. We thus present the analysis efforts on the data from Science Run 2.

Here we provide more details on the analysis procedure. The identification of events likely to be produced by ALP domain-wall crossings comprise three stages: preprocessing, velocity scanning and post-selection^30^. First, in the preprocessing stage, a rolling average and filters are applied to the raw data from the GNOME magnetometer, which are originally recorded by the GPS-synchronized data-acquisition system at the rate of 512 samples per second (ref. ^25^). The rolling average is characterized by a 20 s time constant. Noisy frequency bands are suppressed using a first-order Butterworth high-pass filter at 1.67 mHz together with notch filters corresponding to power-line frequencies of 50 or 60 Hz with a quality factor of 60. These filters are applied forward and backward to remove any phase effects. This limits the observable pulse properties to a frequency region to which all the magnetometers are sensitive. Additionally, it guarantees that the duration of the signal is the same for all the sensors. We note that these filter settings may be changed in future analyses.

The local standard deviation around each point in the magnetometer’s data is determined using an iterative process. Outliers are discarded until the standard deviation of the data in the segment converges. The local standard deviation is calculated taking 100 down-sampled points around each data point.

Additionally, auxiliary measurements have shown that the calibration factors used by each magnetometer to convert raw data into magnetic-field units experience change over time due to, for example, changes in the environmental conditions. Upper limits on the errors in the calibration factor due to such drifts over the course of Science Run 2 have been evaluated, as listed in Extended Data Table 1. Calibration errors result in magnetic-field measurement errors proportional to magnetic field *B*_*j*_. The uncertainty resulting from the calibration error is later used to determine the agreement with the domain-wall model, but not in the magnitude-to-uncertainty ratio estimate resulting from the model, since the calibration error affects the signal and noise in the same way.

Second, at the velocity-scanning stage, data from the individual magnetometers are time-shifted according to different relative velocities between Earth and the ALP domain walls. To sample 97.5% of the velocity probability distribution, a scan of the speeds from 53.7 to 770 km s^–1^ with directions covering the full 4π solid angle is chosen; therefore, the domain walls can take any orientation with respect to the movement of Earth. Note that this distribution considers just the observable perpendicular component of the relative domain-wall velocity and neglects the orbital motion of the Earth around the Sun. For low relative velocities, both time between signals at different magnetometers and signal duration diverge. Therefore, the velocity range is determined by the chosen 97.5% coverage and the maximum relative speed of the domain walls travelling at the Galactic escape speed.

The corresponding time-shifted data along with their local standard deviation estimate are fetched from each magnetometer’s rolling-average full-rate data at the rate of 0.1 samples per second. This reduces the amount of data to process, even though keeping the full timing resolution.

The step size used in the speed scan is chosen so that a single step in speed corresponds to time-shift differences of less than the down-sampled sampling period. For each speed, a lattice of directions covering the full 4π solid angle is constructed. The angular difference between adjacent directions is informed by the sampling rate and speed^30^ such that, as for the speed scan, a single step in direction results in time-shift differences of less than the down-sampled sampling period. With the settings used, the velocity-scanning lattice consists of 1,661 points. This number scales with the cube of the down-sampled sampling rate.

After the time shift, the pulses produced by a domain-wall crossing simultaneously appear as if all the magnetometers were placed at the Earth’s centre. This process results in a time-shifted dataset for each lattice velocity on which *χ*^2^ minimization is performed for each time point to estimate the domain-wall parameters. An ALP domain-wall-crossing direction and magnitude ${{{{\mathcal{B}}}}}_{{{\rm{p}}}}$ with the corresponding *P*-value quantifying the agreement is obtained. The *P*-value is evaluated as the probability of obtaining the given *χ*^2^ value or higher from *χ*^2^ minimization. The *P*-value is calculated using the quadrature sum of the standard deviation of the data and the uncertainty due to drifts in the calibration factors. All the data points in every time-shifted dataset are considered to be potential events, characterized by time, *P*-value, and direction and magnitude ${{{{\mathcal{B}}}}}_{{{\rm{p}}}}$ with their associated uncertainties. The magnitude-to-uncertainty ratio of an event *ζ* is the ratio between ${{{{\mathcal{B}}}}}_{{{\rm{p}}}}$ and its associated uncertainty.

Third, in the post-selection stage, two tests are carried out to check if a potential event is consistent with an ALP domain-wall crossing. The domain-wall model test evaluates if the observed signal amplitudes are consistent with the expected pattern of a domain-wall crossing from any possible direction. It is quantified by the aforementioned *P*-value. The directional-consistency test is based on the angular difference between the estimated domain-wall-crossing direction and the direction of velocity corresponding to the particular time-shifted dataset being analysed. In a real domain-wall-crossing event, these two directions should be aligned.

To evaluate the consistency of a potential event with a domain-wall crossing, we impose thresholds on the *P*-value and the angular difference normalized with respect to the angular spacing of the lattice of velocity points for that speed. The thresholds are chosen to guarantee a detection probability of 97.5% with the minimum possible false-positive probability. The false-positive analysis is performed on the background data. The true-positive analysis is performed on the test data consisting of background data with randomly inserted domain-wall signals as described below.

A single signal pattern may appear as multiple potential events in the analysis, whereas we are seeking to characterize a single underlying domain-wall-crossing event. For example, a signal consistent with a domain-wall crossing lasting for multiple sampling periods would appear as multiple potential events in a single time-shifted dataset. Furthermore, even if such a signal lasts only for a single sampling period, the corresponding potential events appear in different time-shifted datasets. Since it is assumed that domain-wall crossings rarely occur, such clusters of potential events are classified as a single ‘event’. To reduce the double counting of these events, conditions are imposed. If potential events passing the thresholds occur at the same time in different time-shifted datasets or are contiguous in time, the potential event with the greatest magnitude-to-uncertainty ratio is classified as the corresponding single event.

To evaluate the detection probability of the search algorithm, a well-characterized dataset that includes domain-wall-crossing signals with known properties is required. For this purpose, we generate a background dataset by randomly time shuffling the search data so that the relative timing of measurements from different GNOME stations is shifted by amounts so large that no true-positive events could occur. By repeating the process of time shuffling, the length of background data can be made to far exceed the search data. This method is used to generate background data with noise characteristics closely reproducing those of the search data^53^. A set of pseudo-magnetic-field pulses matching the expected amplitude and timing pattern produced by the passages of Earth through the ALP domain walls are inserted into the background data to create the test data.

The true-positive analysis studies the detection probability as a function of the thresholds. Multiple test datasets are created featuring domain-wall-signal patterns with random parameters by inserting Lorentzian-shaped pulses into the background data of the different GNOME magnetometers. The domain-crossing events have magnitudes of ${{{{\mathcal{B}}}}}_{{{\rm{p}}}}$ randomly selected between 0.1 and 10^4^ pT and durations randomly selected between 0.01 and 10^3^ s. The distributions of the these randomized parameters are chosen to be flat on a logarithmic scale. Additionally, the signals are inserted at random times with random directions. To simulate the effects of calibration error, the pulse amplitudes inserted in each magnetometer are weighted by a random factor whose range is given in Extended Data Table 1. The crossing velocity is also randomized within the range covered by the velocity lattice. For each inserted domain-wall-crossing event, the *P-*value, normalized angular difference and magnitude-to-uncertainty ratio are computed.

Extended Data Fig. 2a shows the detection probability as a function of the threshold on the lower limit of the *P-*value and the threshold on the upper limit of the normalized angular difference. We restrict the analysis in Extended Data Fig. 2a to events inserted with a magnitude-to-uncertainty ratio between 5 and 10. This enables a reliable determination of the true-positive detection probability without major contamination by false-positive events, since the background event probability above *ζ* = 5 is below 0.01% in a 10 s sampling interval. Since the detection probability increases with the signal magnitude, we focus on the events below *ζ* = 10. The detection probability is then the number of detected events divided by the number of inserted events. The black line marks the numerically evaluated boundary of the area, guaranteeing at least 97.5% detection. All points along this black line yield the desired detection probability; therefore, this particular choice is made to minimize the number of candidate events when applying the search algorithm to the background data. Here the values determined for the *P*-value threshold and directional-consistency threshold are 0.001 and 3.5, respectively (represented as the white dot in Extended Data Fig. 2a). Extended Data Fig. 2b shows that the detection probability is greater than 97.5% for events featuring a magnitude-to-uncertainty ratio above 5 and guarantees *ϵ* ≥ 95%. This results in an overall detection efficiency of *ϵ* ≥ 95% for the search algorithm, considering both incomplete velocity lattice coverage and detection probability.

Since the noise has a non-zero probability of mimicking the signal pattern expected from an ALP domain-wall crossing well enough to pass the *P*-value and directional-consistency tests, we perform a false-positive study on background data of length *T*_b_. The analysis algorithm is applied to *T*_b_ = 10.7 years of time-shuffled data to establish the rate of events solely expected from the background. Because of the larger amount of background data analysed, lower rates and larger magnitude-to-uncertainty ratios are accessible compared with the search data. Based on the false-positive study, the probability of finding one or more events in the search data above *ζ* is^54^

5 $$P(\ge 1\,\,{{\mbox{above}}}\,\,\zeta )=1-\exp \left(-\frac{T}{{T}_{\mathrm{b}}}\left[1+{n}_{\mathrm{b}}(\zeta )\right]\right),$$

where *T* = 23 days is the duration of Science Run 2 and *n*_b_(*ζ*) is the number of candidate events found in the background data above *ζ*. The significance is then defined as $S=-\sqrt{2}{{{\mathrm{erf}}}\,}^{-1}\left[1-2(1-P)\right]$, where erf^–1^ is the inverse error function. The significance is given in units of the Gaussian standard deviation that corresponds to a one-sided probability of *P*.

After characterizing the background for Science Run 2, the search data are analysed. The results are represented as a solid green line in Fig. 2. For *ζ* > 6, only a few events were found. The event with the largest magnitude-to-uncertainty ratio, *ζ*_max_, was measured at 12.6 followed by additional events at 6.2 and 5.6. From equation (5), the significance associated with finding one or more events produced by the background featuring at least *ζ*_max_ is lower than one sigma. This null result defines the sensitivity of the search and is used to set constraints on the parameter space describing the ALP domain walls.

The observable rate of domain-wall crossings depends on how long GNOME was sensitive to different signal durations and magnitudes. For the evaluation of this effective time, the raw data of each magnetometer are divided into continuous segments between one and two hours depending on the availability of data. The preprocessing steps are applied to each segment. Then, the data are binned by taking the average in 20 s intervals. To estimate the noise in each magnetometer, the standard deviation in each binned segment is calculated to define the covariance matrix *Σ*_s_. The domain-wall magnitude, crossing with the worse-case direction **m**, needed to produce *ζ* = 1 is calculated, as in ref. ^30^, for each bin.

6 $${{{{\mathcal{B}}}}}_{{{{\rm{p}}}}}^{\prime}({{\Delta }}t)=\sqrt{{{{{\bf{m}}}}}{\left({D}_{{{\Delta }}t}^{T}{{{\Sigma }}}_{\mathrm{s}}^{-1}{D}_{{{\Delta }}t}\right)}^{-1}{{{{\bf{m}}}}}},$$

The matrix *D*_Δ*t*_ contains the sensitivity axes of the magnetometers, factor *σ*_p_/*g*, and effects of preprocessing as a function of signal duration (as described in ref. ^30^). Such prepocessing effects rely on a Lorentzian-shaped signal and give rise to the characteristic shape shown in Fig. 3. The effective time *T* is defined as the amount of time for which the network can measure a domain wall with duration Δ*t* and magnitude ${{{{\mathcal{B}}}}}_{{{{\rm{p}}}}}^{\prime}$, producing *ζ* ≥ 1. Monte Carlo simulations analysing segments with inserted domain-wall encounters on the raw data show good agreement with the sensitivity estimation in equation (6).

Assuming that the domain-wall encounters follow Poisson statistics, a bound on the observable rate of events above *ζ*_max_ with 90% confidence is set as^38^

7 $${R}_{90 \% }=\frac{-{{\mathrm{log}}}\,(0.1)}{\epsilon \,{T}({{\Delta }}t,{{{{\mathcal{B}}}}}_{{{{\rm{p}}}}}^{\prime})}.$$

The domain-wall thickness is determined by the ALP mass, and is of the order of the ALP-reduced Compton wavelength *ƛ*_a_ (ref. ^22^):

8 $${{\Delta }}\ x\approx 2\sqrt{2}\,\,{{\lambda}}_{\mathrm{a}}=2\sqrt{2}\frac{\hslash }{{m}_{\mathrm{a}}c}.$$

The constant prefactor of $2\sqrt{2}$ is obtained by approximating the spatial profile of the field-gradient magnitude as a Lorentzian and defining the thickness as the full-width at half-maximum (FWHM). For a given relative-velocity component perpendicular to the domain wall *v*_⊥_, the signal duration is

9 $${{\Delta }}t=\frac{{{\Delta }}\ x}{{v}_{\perp }}\propto {m}_{\rm{a}}^{-1}\,.$$

We assume that domain walls comprise the dominant component of dark matter. Thus, with the energy density *ρ*_DW_ ≈ 0.4 GeV cm^–3^ in the Milky Way^24^, the energy per unit area (surface tension) in a domain wall, *σ*_DW_, determines the average separation between the domain walls, *L*. The surface tension *σ*_DW_ is related to the symmetry-breaking scale^9^ as

10 $${\sigma }_{{{\rm{DW}}}}=\frac{8}{{\hslash }^{2}}{m}_{\rm{a}}{f}_{{{\rm{SB}}}\,}^{2}\,.$$

The average domain-wall separation is then approximated by

11 $${L}\approx \frac{{\sigma }_{{{\rm{DW}}}}}{{\rho }_{{{\rm{DW}}}}}=\frac{8}{{\hslash }^{2}}\frac{{m}_{\mathrm{a}}{f}_{{{\rm{SB}}}}^{2}}{{\rho }_{{{\rm{DW}}}}}\,,$$

which results in the average domain-wall encounter rate of

12 $$r={\bar{v}}/{L}\propto {\left({m}_{\rm{a}}{f}_{{{\rm{SB}}}}^{2}\right)}^{-1}.$$

We assume the typical relative domain-wall speed to be equal to the Galactic rotation speed of Earth.

The ALP parameter space is constrained by imposing *r* ≥ *R*_90%_. The experimental constraint on the coupling constant is written as follows (Supplementary equation (13) in Supplementary Section II).

13 $${f}_{{{\rm{int}}}}\le \frac{\hslash }{\xi }\sqrt{\frac{{\bar{v}}{\rho }_{{{\rm{DW}}}}\epsilon }{8{m}_{\rm{a}}{{\mathrm{log}}}\,(0.1)}{T}({{\Delta }}t,{{{{\mathcal{B}}}}}_{{{{\rm{p}}}}}^{\prime})}$$

The signal duration can be written in terms of the mass of the hypothetical ALP particle and the specific domain-wall-crossing speed, ${{\Delta }}t=\frac{2\sqrt{2}\hslash }{{v}{m}_{\rm{a}}c}$. When calculating the constraints on *f*_int_, we fix the domain-wall-crossing speed to the typical relative speed from the standard halo model, $\bar{v}$  = 300 km s^−1^ (ref. ^23^). In contrast to the signal duration, the pseudo-magnetic-field signal depends on all the parameters of the ALPs, mass, and ratio between the coupling and symmetry-breaking constants, namely, ${{{{\mathcal{B}}}}}_{{{{\rm{p}}}}}^{\prime}=\frac{4{m}_{\rm{a}}{c}^{2}\xi }{{\mu }_{\rm{B}}\zeta }$. The data shown in Fig. 4 are obtained using equation (13) by taking *ζ* = 12.6. The shape of the constrained space is given by the fact that *T* varies depending on the target *m*_a_ and *ξ*.

## Online content

Any methods, additional references, Nature Research reporting summaries, source data, extended data, supplementary information, acknowledgements, peer review information; details of author contributions and competing interests; and statements of data and code availability are available at 10.1038/s41567-021-01393-y.

### Supplementary information

Supplementary Information Supplementary text.

### Source data

Source Data Fig. 2 Positive event data (Fig. 2).

Source Data Fig. 3 Time for which GNOME was sensitive to Lorentzian signals of various FWHM values and magnitudes (Fig. 3).

Source Data Fig. 4 Bounds on ALP parameter space (Fig. 4).

Source Data Extended Data Fig. 1 Noise data from each magnetometer (Extended Data Fig. 1).

## Figures and Tables

**Extended Data Table 1:**
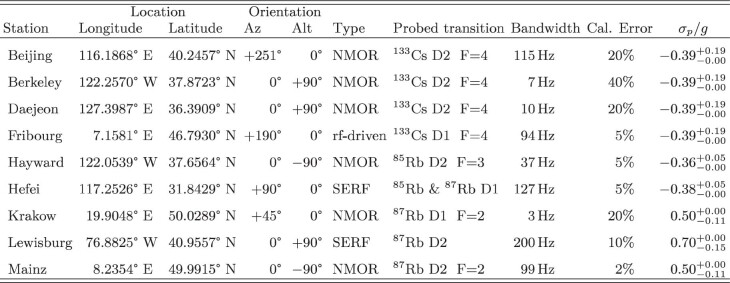
Characteristics of the magnetometers active during Science Run 2. The station name, location in longitude and latitude, orientation of the sensitive axis, type of magnetometer (NMOR^50,51^, rf-driven^26^, or SERF^52^), and probed transition are listed. The bandwidth indicates the measured -3 dB point of the magnetometers’ frequency response to oscillating magnetic fields. The calibration error takes into account potential temporal variation of the magnetometers’ calibration over the course of Science Run 2, and is estimated based on auxiliary measurements. The rightmost column lists the estimated ratio between the effective proton spin polarization and the Landé g-factor for the magnetometer, ${\sigma_{\rm{p}}/{g}}$, which depends on the atomic species and the magnetometry scheme as described in Sec. II of the Supplementary Information. The ${\sigma_{\rm{p}}/{g}}$ value is used to relate the measured magnetic field to the signal expected from the interaction of an ALP field with proton spins. The indicated uncertainty describes the range of values from different theoretical calculations^29^

## Data Availability

Source data are provided with this paper. The datasets and analysis code used in the current study are available from the corresponding authors upon reasonable request. Also, see the collaboration website https://budker.uni-mainz.de/gnome/ where all the available data are displayed.

## Code availability

The code used in the current study is available from the corresponding authors upon reasonable request.
