# Supplementary material for: Search for topological defect dark matter with a global network of optical magnetometers
Source: Nat Phys. 2021 Dec 7;17(12):1396–401. doi: 10.1038/s41567-021-01393-y (PMC8654677; doi:10.1038/s41567-021-01393-y)
Supplement: Supplementary file 1 — Supplementary text. [file 41567_2021_1393_MOESM1_ESM.pdf]

nature portfolio

https://doi.org/10.1038/s41567-021-01393-y

Supplementary information

Search for topological defect dark matter with a global network of optical magnetometers

In the format provided by the
authors and unedited

Supplementary Information

I. RELATING MEASURED PARAMETERS TO ALP DOMAIN-WALL PARAMETER SPACE

Domain walls form when a field can monotonically vary across vacuum states; two degenerate vacua or possibly the same state if, for example, the field takes values on the 1-sphere. This is the case for ALPs which arise from the angular part of a complex scalar field [1].

The following Lagrangian terms are considered in natural units ($\hbar = c = 1$ here and throughout the supplementary information) for a new complex scalar field ϕ,

$$\mathcal{L} \supset |\partial_\mu \phi|^2 - \frac{\lambda}{S_0^{2N-4}} |2^{N/2}\phi^N + S_0^N|^2 \;, \tag{SI.1}$$

where λ is a unitless constant and S_0 is a constant with units of energy [2]. Other references may use a minus sign in front of the S_0^N term, which results in a similar potential, up to a phase. The end result of Eq. (SI.1) is that the axion potential will have a maximum at zero, while the minus-convention will have a vacuum at zero.

The axion field is obtained by reparameterizing the complex field ϕ in Eq. (SI.1) in terms of the real field S (Higgs) and a (axion),

$$\phi = \frac{S}{\sqrt{2}} \exp(ia/S_0) \;.$$

The second term in Eq. (SI.1) will break the $U(1)$ symmetry of the complex field into a discrete $\mathbb{Z}_N$ symmetry, $\phi \to \exp(2\pi i k/N)\phi$ for integer k. This corresponds to the axion shift-symmetry, $a \to a + \frac{2\pi S_0}{N} k$. The Higgs mode obtains a vacuum expectation value $S \to S_0$, and the axion field has degenerate vacua or ground energy states at $a = \frac{\pi S_0}{N}(2k+1)$ for integer k. One can define the symmetry-breaking scale as $f_{\rm SB} = S_0/N$. Reparameterizing the complex scalar field in Eq. (SI.1) and setting the Higgs mode to the vacuum expectation value, the axion Lagrangian is

$$\mathcal{L}_a = \frac{1}{2}(\partial_\mu a)^2 - 2m_a^2 f_{\rm SB}^2 \cos^2\left(\frac{a}{2f_{\rm SB}}\right) \;, \tag{SI.2}$$

where the axion mass is $m_a = N S_0 \sqrt{2\lambda}$. This can be seen by matching the second-derivative of the cosine term at the minima to a scalar mass term.

For simplicity, a static domain wall in the yz-plane separating domains of $-\pi f_{\rm SB}$ and $+\pi f_{\rm SB}$ is considered. Solving the classical field equations, one finds

$$a(x) = 2f_{\rm SB} \arcsin\left[\tanh(m_a x)\right] \;. \tag{SI.3}$$

The gradient of the field is then

$$\frac{da}{dx}(x) = \frac{2f_{\rm SB} m_a}{\cosh(m_a x)} \;. \tag{SI.4}$$

This has the full width at half maximum,

$$\Delta x = \frac{2\cosh^{-1}(2)}{m_a} \approx \frac{2\sqrt{2}}{m_a} \;. \tag{SI.5}$$

Using the domain-wall solution [Eq. (SI.3)] and integrating the energy density of the domain wall over x yields the surface tension (energy per unit area) [2],

$$\sigma_{\rm DW} = 8 m_a f_{\rm SB}^2 \;. \tag{SI.6}$$

Interactions observable by magnetometers involve coupling between the axion field gradient and the axial-vector current of a fermionic field. For a fermion field ψ, the interaction is

$$\begin{aligned} \mathcal{L}_{\rm int} =& \frac{i(\phi\partial_\mu\phi^* - \partial_\mu\phi\phi^*)}{S_0 f_{\rm int}} \bar{\psi}\gamma^\mu\gamma^5\psi \\ &\xrightarrow{S\to S_0} \frac{\partial_\mu a}{f_{\rm int}} \bar{\psi}\gamma^\mu\gamma^5\psi \;. \end{aligned} \tag{SI.7}$$

The axial-vector current is related to the spin $\boldsymbol{S}$, so that the interaction Hamiltonian becomes

$$H_{\text{int}} = \frac{1}{f_{\text{int}}} \boldsymbol{\nabla} a \cdot \frac{\boldsymbol{S}}{\|S\|} \ , \tag{SI.8}$$

i.e., for spin-½ particles, $1/\|S\| = 2$.

Optical magnetometers operate by measuring the change in atomic energy levels between two energy states with magnetic quantum numbers differing by Δm_F. The sensitive axis of a magnetometer is either defined by a leading magnetic field applied to the atoms, or, in the case of zero-field magnetometers, by the axis orthogonal to the plane defined by the propagation directions of the pump and probe light. Variations of the magnetic field along the sensitive axis are measured. The spin coupling from Eq. (SI.8) can induce a similar shift in energy levels to a magnetic field. The maximum energy shift is determined by plugging the largest gradient from Eq. (SI.4) into Eq. (SI.8),

$$\Delta E = \sum_{i \in \text{e,p,n},...} \frac{2\eta \sigma_{(i)} \Delta m_F}{\|S_{(i)}\|} \frac{f_{\text{SB}}}{f_{\text{int}}^{(i)}} m_a \ , \tag{SI.9}$$

where i labels the species of fermion, $\sigma_{(i)} = \frac{\langle \boldsymbol{S}_{(i)} \cdot \boldsymbol{F}_{(i)} \rangle}{\boldsymbol{F}_{(i)}^2}$ is the projected spin coupling, $\eta = \cos\theta$ for the angle between the axion gradient and sensitive axis θ, and $f_{\text{int}}^{(i)}$ is the interaction coupling to particle i. In general, we will combine the $\frac{\sigma_{(i)}}{\|S_{(i)}\| f_{\text{int}}^{(i)}}$ terms into an effective ratio $\frac{2\sigma_j}{f_{\text{int}}}$, where j now labels the magnetometer. Comparing this to the energy shift due to a magnetic field, $\Delta E_B = g_{F,j} \mu_B \Delta m_F B_j$, one obtains a relationship for a normalized pseudo-magnetic field,

$$\frac{g_{F,j} B_j}{\sigma_j \eta_j} = \frac{4}{\mu_B} m_a \xi \equiv \mathcal{B}_{\text{p}} \ , \tag{SI.10}$$

for $\xi \equiv \frac{f_{\text{SB}}}{f_{\text{int}}}$, $g_{F,j}$ being the g-factor for the magnetometer j, and $\|S_{(i)}\| = 1/2$ since we only consider coupling to spin-½ particles. Here, the normalization is such that $\mathcal{B}_{\text{p}}$ is the same for all magnetometers, though each individual sensor may observe a different pseudo-magnetic field, B_j.

There are two factors that must be considered when determining if axion domain walls are observable by the network: the magnitude of the signal $\mathcal{B}_{\text{p}}$ and the rate of signals. Domain walls are assumed to exist in a static (or virialized) network across the galaxy through which Earth traverses. For a domain-wall velocity v, the duration of a signal is $\Delta t = \Delta x / v$. Filters and bandwidth limitations generally reduce the magnitude by a factor dependent on the signal duration, which affects the sensitivity of the network (see Appendix in Ref. [3]).

Meanwhile, if the domain walls induce a strong enough signal to be observed, but are so infrequent that one is unlikely to be found over the course of a measurement, then the network is effectively insensitive. If the energy density of domain walls across the galaxy is ρ_{DW}, then the average rate of domain walls passing through Earth is given by

$$r = \frac{\bar{v} \rho_{\text{DW}}}{\sigma_{\text{DW}}} = \frac{\bar{v} \rho_{\text{DW}}}{8 m_a f_{\text{SB}}^2} \ , \tag{SI.11}$$

where $\bar{v}$ is the typical relative speed.

The physical parameters describing the ALP domain walls (m_a, f_{SB}, and f_{int}) must be related to the parameters observable by the network ($\mathcal{B}_{\text{p}}$ and Δt). The energy density ρ_{DW} and the typical relative speed $\bar{v}$ are assumed according to the observed dark matter energy density and the galactic rotation velocity, respectively.

In order to determine if a set of physical parameters is observable, the likelihood that no events are found must be constrained. This constraint defines the confidence level of the detection. The probability of observing k events given that one expects to observe μ events is given by the Poisson probability mass function,

$$P(k; \mu) = \frac{\mu^k}{k!} e^{-\mu} \ .$$

However, the network also has some detection efficiency $\epsilon < 1$, so there could be multiple domain-wall-crossing events, but no detection. In particular, the chance of missing no events given that there were k events is $(1-\epsilon)^k$. For an event rate r and measurement time T, the probability that no events are detected is then

$$\sum_{k=0}^{\infty} (1-\epsilon)^k \frac{(rT)^k}{k!} e^{-rT} = e^{-\epsilon r T} \ .$$

A bound on the event rate R_C at confidence level C is then given by demanding the probability of observing at least one event $1 - e^{-\epsilon R_C T} \geq C$, likewise, one would expect to observe event rates

$$r \geq R_C \equiv \frac{-\log(1-C)}{\epsilon T} \ . \tag{SI.12}$$

The physical parameter space of the ALPs is constrained by demanding that $r \geq R_C$. Similar arguments for defining constraints can be found, e.g., Ref. [4]. The total time that the network is sensitive to the measurable parameters, $\tilde{T}(\Delta t, \mathcal{B}'_{\rm p})$, may be less than the total measurement time. These parameters are related to the physical parameters via Eq. (SI.5) and Eq. (SI.10). One finds a sensitivity bound for $f_{\rm int}$ in terms of m_a and ξ,

$$f_{\rm int} \leq \frac{1}{\xi}\sqrt{\frac{-\bar{v}\rho_{\rm DW}\epsilon}{8m_a \log(1-C)}\tilde{T}\left(\Delta t, \mathcal{B}'_{\rm p}\right)} \tag{SI.13}$$
$$\text{for} \quad \Delta t = \frac{2\sqrt{2}}{\bar{v}m_a} \quad \text{and} \quad \mathcal{B}'_{\rm p} = \frac{4m_a\xi}{\mu_B\zeta} \ ,$$

where $\mathcal{B}'_{\rm p}$ is the sensitivity of the network and ζ is the magnitude-to-noise ratio induced by a signal with magnitude $\mathcal{B}_{\rm p}$. The main calculation from the network data needed for this sensitivity bound is $\tilde{T}$. This is calculated by measuring the sensitivity of the network over time for different signal durations Δt and integrating over the time during which the network is sensitive to $\mathcal{B}_{\rm p} = \zeta\mathcal{B}'_{\rm p}$. Finally, if, after analyzing the data, no domain walls are found, Eq. (SI.13) defines an exclusion region.

II. CONVERSION BETWEEN MAGNETIC FIELD UNITS AND PROTON SPIN COUPLING

The amplitude of a signal appearing in the magnetic field data from a GNOME magnetometer due to interaction of atomic spins with an ALP field a via the linear coupling described by Eq. (1) varies based on the atomic species. In every GNOME magnetometer, the atomic vapor cell is located within a multi-layer magnetic shield made of mu-metal and, in some cases a ferrite innermost layer. Interactions of the ALP field with electron spins in the magnetic shielding material can generate a compensating magnetic field that could partially cancel the energy shift due to the interaction of the ALP field with atomic electrons in the vapor cell, as discussed in Ref. [5]. For this reason, GNOME magnetometers are most sensitive to interactions of the ALP field with nuclear spins.

A. Deriving spin-projection

All GNOME magnetometers active during Science Run 2 measure spin-dependent interactions of alkali atoms whose nuclei have valence protons. Thus the GNOME is primarily sensitive to spin-dependent interactions of ALP fields with proton spins. Consequently, the expected signal amplitude measured by a GNOME magnetometer due to the pseudo-magnetic field pulse from passage of Earth through an ALP domain wall must be rescaled by the ratio of the proton spin content of the probed ground-state hyperfine level(s) to their gyromagnetic ratio. Some GNOME magnetometers optically pump and probe a single ground-state hyperfine level, while others rely on the technique of spin-exchange relaxation free (SERF) magnetometry in which the spin-exchange collision rate is much faster than the Larmor precession frequency [6–8]. For SERF magnetometers a weighted average of the ground-state Zeeman sublevels over both ground-state hyperfine levels is optically pumped and probed.

Table SI.1 shows the relevant factors needed to convert the magnetic field signal recorded by GNOME magnetometers into the expected pseudo-magnetic field due to interaction of an ALP field with the proton spin. Detailed calculations are carried out in Ref. [15]. The relationship of the expectation value for total atomic angular momentum $\langle \mathbf{F} \rangle$ to the nuclear spin $\langle \mathbf{I} \rangle$ can be estimated based on the Russell-Saunders LS-coupling scheme:

$$\begin{aligned} \langle \mathbf{F} \rangle &= \langle \mathbf{S}_e \rangle + \langle \mathbf{L} \rangle + \langle \mathbf{I} \rangle \ , \\ &= \frac{\langle \mathbf{S}_e \cdot \mathbf{F} \rangle}{F(F+1)}\langle \mathbf{F} \rangle + \frac{\langle \mathbf{L} \cdot \mathbf{F} \rangle}{F(F+1)}\langle \mathbf{F} \rangle + \frac{\langle \mathbf{I} \cdot \mathbf{F} \rangle}{F(F+1)}\langle \mathbf{F} \rangle \ , \end{aligned} \tag{SI.14}$$

where $\mathbf{S}_e$ is the electronic spin and $\mathbf{L}$ is the orbital angular momentum. GNOME magnetometers pump and probe atomic states with $L = 0$, which simplifies the above equation to:

$$\langle \mathbf{F} \rangle = \frac{\langle \mathbf{S}_e \cdot \mathbf{F} \rangle}{F(F+1)}\langle \mathbf{F} \rangle + \frac{\langle \mathbf{I} \cdot \mathbf{F} \rangle}{F(F+1)}\langle \mathbf{F} \rangle \ . \tag{SI.15}$$

Table SI.1. Fractional proton spin polarization $\sigma_{p,F}$, Landé g-factors g_F, and their ratios for the ground state hyperfine levels used in GNOME, and the weighted average of these values across both hyperfine levels ($\langle\sigma_p\rangle_{\rm hf}/\langle g\rangle_{\rm hf}$) applicable to SERF magnetometers in the low-spin-polarization limit. The estimates are based on the single-particle Schmidt model for nuclear spin [9] and the Russell-Saunders scheme for the atomic states. Uncertainties in the values for $\sigma_{p,F}$ describe the range of different results from calculations based on the Schmidt model, semi-empirical models [10, 11], and large-scale nuclear shell model calculations where available [12–14]. The uncertainties in $\sigma_{p,F}$ and $\langle\sigma_p\rangle_{\rm hf}$ are one-sided because alternative methods to the Schmidt model generally predict smaller absolute values of the proton spin polarization. See Ref. [15] for further details.

Atom (state)	$\sigma_{p,F}$	g_F	$\sigma_{p,F}/g_F$	$\langle\sigma_p\rangle_{\rm hf}/\langle g\rangle_{\rm hf}$
^{39}K ($F=2$)	$-0.15^{+0.06}_{-0.00}$	0.50	$-0.30^{+0.12}_{-0.00}$	$-0.5^{+0.2}_{-0.0}$
^{39}K ($F=1$)	$-0.25^{+0.10}_{-0.00}$	-0.50	$0.50^{+0.00}_{-0.19}$	
^{85}Rb ($F=3$)	$-0.12^{+0.02}_{-0.00}$	0.33	$-0.36^{+0.05}_{-0.00}$	$-0.8^{+0.1}_{-0.0}$
^{85}Rb ($F=2$)	$-0.17^{+0.02}_{-0.00}$	-0.33	$0.50^{+0.00}_{-0.07}$	
^{87}Rb ($F=2$)	$0.25^{+0.00}_{-0.05}$	0.50	$0.50^{+0.00}_{-0.11}$	$0.8^{+0.0}_{-0.2}$
^{87}Rb ($F=1$)	$0.42^{+0.00}_{-0.09}$	-0.50	$-0.83^{+0.18}_{-0.00}$	
^{133}Cs ($F=4$)	$-0.10^{+0.05}_{-0.00}$	0.25	$-0.39^{+0.19}_{-0.00}$	$-1.2^{+0.6}_{-0.0}$
^{133}Cs ($F=3$)	$-0.13^{+0.06}_{-0.00}$	-0.25	$0.50^{+0.00}_{-0.24}$	

For $L=0$ the projection of $\mathbf{I}$ on $\mathbf{F}$ is given by

$$\langle \mathbf{I}\cdot\mathbf{F}\rangle = \frac{1}{2}\left[F(F+1)+I(I+1)-S_e(S_e+1)\right] . \tag{SI.16}$$

The above relations define the fractional spin polarization of the nucleus relative to the spin polarization of the atom:

$$\sigma_{N,F} \equiv \frac{\langle \mathbf{I}\cdot\mathbf{F}\rangle}{F(F+1)} . \tag{SI.17}$$

The next step is to relate $\sigma_{N,F}$ to the spin polarization of the valence proton $\sigma_{p,F}$ for a particular F. As discussed in Ref. [15], a reasonable estimate for K, Rb, and Cs nuclei can be obtained from the nuclear shell model by assuming that the nuclear spin $\mathbf{I}$ is due to the orbital motion and intrinsic spin of only the valence nucleon and that the spin and orbital angular momenta of all other nucleons sum to zero. This is the assumption of the Schmidt or single-particle model [9]. In the Schmidt model, the nuclear spin $\mathbf{I}$ is generated by a combination of the valence nucleon spin ($\mathbf{S}_p$) and the valence nucleon orbital angular momentum $\boldsymbol{\ell}$, so that we have

$$\begin{aligned}\sigma_{p,F} &= \frac{\langle \mathbf{S}_p\cdot\mathbf{I}\rangle}{I(I+1)}\sigma_{N,F} ,\\ &= \frac{S_p(S_p+1)+I(I+1)-\ell(\ell+1)}{2I(I+1)}\sigma_{N,F} ,\\ &= \sigma_p\sigma_{N,F} ,\end{aligned} \tag{SI.18}$$

where it is assumed that the valence nucleon is in a well-defined state of ℓ and S_p, and σ_p is defined to be the fractional proton spin polarization for a given nucleus [15].

For comparison between GNOME magnetometers using different atomic species, it is essential to evaluate the uncertainty in the estimate of $\sigma_{p,F}$ based on the Schmidt model. To estimate this uncertainty, we compare calculations of $\sigma_{p,F}$ based on the Schmidt model to the results of the semi-empirical calculations described in Refs. [10, 11] and to the results of detailed nuclear shell-model calculations where available [12–14]. Conservatively, we assign the uncertainty in $\sigma_{p,F}$ to be given by the full range (maximum to minimum) of the values of $\sigma_{p,F}$ calculated by these various methods. It turns out that in each considered case, the estimate based on the Schmidt model gives the largest value of $|\sigma_{p,F}|$, causing the theoretical uncertainties in estimates of $\sigma_{p,F}$ to be one-sided as shown in Table SI.1. Further details are discussed in Ref. [15].

B. SERF magnetometers

SERF magnetometers operate in a regime where the Larmor frequency is small compared to the spin-exchange rate, so that the rapid spin-exchange locks together the expectation values of the angular momentum projection $\langle M_F \rangle$ in both ground-state hyperfine levels of the alkali atom. Because the Landé g-factors g_F in the two ground-state hyperfine levels have nearly equal magnitudes but opposite signs, the magnitude of the effective Landé g-factor in a SERF magnetometer, $\langle g \rangle_{\rm hf}$, is reduced compared to that in optical atomic magnetometers where a single ground-state hyperfine level is probed.

To calculate the effective Landé g-factor averaged over hyperfine levels, $\langle g \rangle_{\rm hf}$, for a SERF magnetometer, it is instructive to consider the equation describing the magnetic torque on an alkali atom,

$$g_s \mu_B \mathbf{B} \times \langle \mathbf{S}_e \rangle \approx \frac{d\langle \mathbf{F} \rangle}{dt} \ , \tag{SI.19}$$

where $g_s \approx 2$ is the electron g-factor and we have ignored the contribution of the nuclear magnetic moment. In the SERF regime, where the alkali vapor is in spin-exchange equilibrium, the populations of the Zeeman sublevels correspond to the spin-temperature distribution [16] described by a density matrix in the Zeeman basis given by [17, 18]

$$\boldsymbol{\rho} = C e^{\boldsymbol{\beta} \cdot \mathbf{F}} \ , \tag{SI.20}$$

where C is a normalization constant and $\boldsymbol{\beta}$ is the spin-temperature vector defined to point in the direction of the spin polarization P with magnitude

$$\beta = \ln\left(\frac{1+P}{1-P}\right) \ . \tag{SI.21}$$

In the low-spin-polarization limit,

$$\boldsymbol{\rho} \approx C(1 + \boldsymbol{\beta} \cdot \mathbf{F}) \ . \tag{SI.22}$$

If follows that

$$\langle \mathbf{S}_e \rangle = \mathrm{Tr}(\boldsymbol{\rho} \mathbf{S}_e) = \frac{1}{3} S_e(S_e+1)\boldsymbol{\beta} = \frac{1}{4}\boldsymbol{\beta} \ , \tag{SI.23}$$

and

$$\langle \mathbf{I} \rangle = \mathrm{Tr}(\boldsymbol{\rho} \mathbf{I}) = \frac{1}{3} I(I+1)\boldsymbol{\beta} \ . \tag{SI.24}$$

Substituting the above expressions into Eq. (SI.19) yields

$$\langle g \rangle_{\rm hf} \mu_B \mathbf{B} \times \boldsymbol{\beta} \approx \frac{d\boldsymbol{\beta}}{dt} \ , \tag{SI.25}$$

where

$$\langle g \rangle_{\rm hf} = \frac{3 g_s}{3 + 4I(I+1)} \ . \tag{SI.26}$$

Equation (SI.26) can be compared to the g-factor for a particular alkali ground-state hyperfine level [19],

$$g_F = \pm \frac{g_s}{2I+1} \ . \tag{SI.27}$$

The effective proton spin polarization $\langle \sigma_p \rangle_{\rm hf}$ for SERF magnetometers can also be derived by considering the relevant torque equation

$$\begin{aligned} \left(\frac{1}{f_{\rm int}} \boldsymbol{\nabla} a\right) \times \frac{\langle \mathbf{S}_p \rangle}{\| S_p \|} &= \frac{d\langle \mathbf{F} \rangle}{dt} \ , \\ \left(\frac{1}{f_{\rm int}} \boldsymbol{\nabla} a\right) \times \frac{\sigma_p}{\| S_p \|} \langle \mathbf{I} \rangle &= \frac{d\langle \mathbf{F} \rangle}{dt} \ , \end{aligned} \tag{SI.28}$$

where we have used the fact that GNOME as configured for Science Run 2 is sensitive to the coupling of the ALP field to proton spins. In the low-spin-polarization limit, based on Eqs. (SI.23) and (SI.24),

$$\left(\frac{1}{f_{\rm int}}\boldsymbol{\nabla}a\right)\times\frac{\langle\sigma_p\rangle_{\rm hf}}{\|S_p\|}\boldsymbol{\beta}=\frac{d\boldsymbol{\beta}}{dt}\ , \tag{SI.29}$$

where

$$\langle\sigma_p\rangle_{\rm hf}=\sigma_p\frac{4I(I+1)}{3+4I(I+1)}\ . \tag{SI.30}$$

Table SI.1 shows the ratio between the effective proton spin polarization averaged over both ground-state hyperfine levels, $\langle\sigma_p\rangle_{\rm hf}$, to the effective Landé g-factor, $\langle g\rangle_{\rm hf}$, in the low-spin-polarization regime. Note that the magnitudes of $\langle\sigma_p\rangle_{\rm hf}/\langle g\rangle_{\rm hf}$ are in general similar or slightly larger than the magnitudes of $\sigma_{p,F}/g_F$ for a single hyperfine level.

To determine the actual values of $\langle\sigma_p\rangle_{\rm hf}/\langle g\rangle_{\rm hf}$ for the SERF magnetometers used in GNOME's Science Run 2, more detailed considerations are required.

The values for the ratio of the effective proton spin polarization to the effective Landé g-factors for each GNOME magnetometer active during Science Run 2 are given in Extended Data Table 1.

1. *Hefei magnetometer*

The Hefei GNOME station employs a SERF magnetometer in a closed-loop, single-beam configuration, where the laser light is resonant with the Rb D1 line (pressure-broadened by 600 torr of nitrogen gas to a linewidth of ~ 10 GHz). The Hefei SERF magnetometer operates in the low-spin-polarization mode. The vapor cell contains ^{39}K, ^{85}Rb, and ^{87}Rb atoms in natural abundance, so spin-exchange collisions average over both ground-state hyperfine levels of all three species. Taking into account the relative abundances of the different atomic species at the cell temperature of ≈ 150°C ($\approx 9\%$ ^{39}K, $\approx 65.5\%$ ^{85}Rb, $\approx 25.5\%$ ^{87}Rb), we find that $\langle g\rangle_{\rm hf}\approx 0.193$, $\langle\sigma_p\rangle_{\rm hf}=-0.073^{+0.010}_{-0.000}$, and thus $\langle\sigma_p\rangle_{\rm hf}/\langle g\rangle_{\rm hf}=-0.38^{+0.05}_{-0.00}$ for the Hefei magnetometer.

2. *Lewisburg magnetometer*

The Lewisburg GNOME station employs a SERF magnetometer in a closed-loop, two-beam configuration. The vapor cell contains only ^{87}Rb atoms. The Lewisburg SERF magnetometer operates with a spin-polarization $P\approx 0.5$, outside the low-spin-polarization regime. Discussions of the high-polarization regime are given in Refs. [18, 20]. For a nucleus with $I=3/2$, the effective Landé g-factor is given by

$$\langle g\rangle_{\rm hf}=g_s\frac{1+P^2}{6+2P^2}\ , \tag{SI.31}$$

and the effective proton spin polarization is given by

$$\langle\sigma_p\rangle_{\rm hf}=\sigma_p\frac{5+P^2}{6+2P^2}\ . \tag{SI.32}$$

Based on Eqs. (SI.31) and (SI.32), we find that for the Lewisburg magnetometer $\langle\sigma_p\rangle_{\rm hf}/\langle g\rangle_{\rm hf}=0.70^{+0.00}_{-0.15}$.

---

[1] Peccei, R. D. & Quinn, H. R. CP conservation in the presence of instantons. *Phys. Rev. Lett.* **38**, 1440–1443 (1977).
[2] Pospelov, M. *et al.* Detecting domain walls of axionlike models using terrestrial experiments. *Phys. Rev. Lett.* **110**, 021803 (2013).
[3] Masia-Roig, H. *et al.* Analysis method for detecting topological defect dark matter with a global magnetometer network. *Phys. Dark Universe* **28**, 100494 (2020).
[4] Brady, P. R., Creighton, J. D. E. & Wiseman, A. G. Upper limits on gravitational-wave signals based on loudest events. *Class. Quantum Grav.* **21**, S1775–S1781 (2004).
[5] Jackson Kimball, D. F. *et al.* Magnetic shielding and exotic spin-dependent interactions. *Phys. Rev. D* **94**, 082005 (2016).
[6] Allred, J., Lyman, R., Kornack, T. & Romalis, M. V. High-sensitivity atomic magnetometer unaffected by spin-exchange relaxation. *Phys. Rev. Lett.* **89**, 130801 (2002).

[7] Kominis, I., Kornack, T., Allred, J. & Romalis, M. V. A subfemtotesla multichannel atomic magnetometer. *Nature* **422**, 596–599 (2003).

[8] Ledbetter, M., Savukov, I., Acosta, V., Budker, D. & Romalis, M. Spin-exchange-relaxation-free magnetometry with Cs vapor. *Phys. Rev. A* **77**, 033408 (2008).

[9] Schmidt, T. Über die magnetischen momente der atomkerne. *Z. Phys.* **106**, 358–361 (1937).

[10] Engel, J. & Vogel, P. Spin-dependent cross sections of weakly interacting massive particles on nuclei. *Phys. Rev. D* **40**, 3132–3135 (1989).

[11] Flambaum, V. & Tedesco, A. Dependence of nuclear magnetic moments on quark masses and limits on temporal variation of fundamental constants from atomic clock experiments. *Phys. Rev. C* **73**, 055501 (2006).

[12] Engel, J., Ressell, M., Towner, I. & Ormand, W. Response of mica to weakly interacting massive particles. *Phys. Rev. C* **52**, 2216–2221 (1995).

[13] Toivanen, P., Kortelainen, M., Suhonen, J. & Toivanen, J. Large-scale shell-model calculations of elastic and inelastic scattering rates of lightest supersymmetric particles (LSP) on ^{127}I, ^{129}Xe, ^{131}Xe, and ^{133}Cs nuclei. *Phys. Rev. C* **79**, 044302 (2009).

[14] Iachello, F., Krauss, L. M. & Maino, G. Spin-dependent scattering of weakly interacting massive particles in heavy nuclei. *Physics Letters B* **254**, 220–224 (1991).

[15] Jackson Kimball, D. F. Nuclear spin content and constraints on exotic spin-dependent couplings. *New J. Phys.* **17**, 073008 (2015).

[16] Anderson, L. W., Pipkin, F. M. & Baird Jr, J. C. N^{14} - N^{15} hyperfine anomaly. *Phys, Rev.* **116**, 87–98 (1959).

[17] Happer, W. & Tang, H. Spin-exchange shift and narrowing of magnetic resonance lines in optically pumped alkali vapors. *Phys. Rev. Lett.* **31**, 273–276 (1973).

[18] Savukov, I. & Romalis, M. Effects of spin-exchange collisions in a high-density alkali-metal vapor in low magnetic fields. *Phys. Rev. A* **71**, 023405 (2005).

[19] Budker, D., Kimball, D. F. & DeMille, D. P. *Atomic physics: an exploration through problems and solutions* (Oxford University Press, USA, 2008).

[20] Appelt, S. *et al.* Theory of spin-exchange optical pumping of ^{3}He and ^{129}Xe. *Phys. Rev. A* **58**, 1412–1439 (1998).
